# Bafilomycin A1 inhibits SARS-CoV-2 infection in a human lung xenograft mouse model

**DOI:** 10.1186/s12985-023-01971-x

**Published:** 2023-01-31

**Authors:** Cuiling Zhang, Bingjie Wei, Zirui Liu, Wei Yao, Yiquan Li, Jing Lu, Chenchen Ge, Xiaoyang Yu, Dapeng Li, Yilong Zhu, Chao Shang, Ningyi Jin, Xiao Li

**Affiliations:** 1 Changchun Veterinary Research Institute, Chinese Academy of Agricultural Sciences, Yujinxiang road, 573, Jingyue Economic and Technological Development Zone, Changchun, 130122 Jilin People’s Republic of China; 2 Veterinary Medicine College, Northwest A&F University, Shaanxi, 712100 People’s Republic of China; 3 Healthcare Department, Agency for Offices Administration, 23 Xinwai Street, Haidian District, Beijing, 100082 People’s Republic of China; 4 Changchun University of Chinese Medicine, Changchun, People’s Republic of China; 5 Jiangsu Co-innovation Center for Prevention and Control of Important Animal Infectious Diseases and Zoonoses, Yangzhou, People’s Republic of China

**Keywords:** Bafilomycin A1, Animal model, SARS-CoV-2, Variants

## Abstract

Coronavirus disease 2019 is a global pandemic caused by SARS-CoV-2. The emergence of its variant strains has posed a considerable challenge to clinical treatment. Therefore, drugs capable of inhibiting SARS-CoV-2 infection, regardless of virus variations, are in urgently need. Our results showed that the endosomal acidification inhibitor, Bafilomycin A1 (Baf-A1), had an inhibitory effect on the viral RNA synthesis of SARS-CoV-2, and its Beta and Delta variants at the concentration of 500 nM. Moreover, the human lung xenograft mouse model was used to investigate the anti-SARS-CoV-2 effect of Baf-A1. It was found that Baf-A1 significantly inhibited SARS-CoV-2 replication in the human lung xenografts by in situ hybridization and RT-PCR assays. Histopathological examination showed that Baf-A1 alleviated SARS-CoV-2-induced diffuse inflammatory infiltration of granulocytes and macrophages and alveolar endothelial cell death in human lung xenografts. In addition, immunohistochemistry analysis indicated that Baf-A1 decreased inflammatory exudation and infiltration in SARS-CoV-2-infected human lung xenografts. Therefore, Baf-A1 may be a candidate drug for SARS-CoV-2 treatment.

## Introduction

The coronavirus disease emerged in late 2019 and rapidly spread into a global pandemic, posing a significant threat to human health. A novel coronavirus was identified as the pathogen causing the outbreak of atypical pneumonia. The World Health Organization (WHO) has given it a nomenclature of 2019 novel coronavirus (2019-nCoV) and declared this outbreak as a global public health emergency on 30 January 2020 [1]. Severe acute respiratory syndrome coronavirus 2 (SARS-CoV-2) is the ninth documented coronavirus that infects humans and the seventh identified in the last 20 years [2]. All previous human coronaviruses have zoonotic origins, similar to most human viruses [3].

SARS-CoV-2 is a member of the *Coronaviridae* family of positive single-stranded RNA viruses [4, 5] and is the third coronavirus known to cause severe acute respiratory disease in humans. The virus has mutated to Alpha (B.1.1.7), Beta (B.1.351), Gamma (P.1), Epsilon (B.1.427), Delta (B.1.617.2) [6, 7], and Omicron (B.1.1.529) [8]. A total of seven variants have been classified as “variants of concern” by the WHO, which brought new control and prevention challenges. The WHO has outlined three key criteria to designate the Delta Variant of Concern (VOC): (1) increased transmissibility, (2) increased virulence, and (3) decreased effectiveness of available diagnostics, vaccines, and therapeutics [9]. However, only a few effective small-molecule drugs are approved for the prevention and treatment of the disease; therefore, drugs capable of effectively treating this infection are urgently needed.

It is known that the acidic pH in endosomes is critical for most viral infections, where the vacuolar H^+^-ATPase (V-ATPase) is a key player and functions as an electrogenic H^+^ pump that translocates protons across membranes to decrease the endomembrane pH [10]. Bafilomycin A1 (Baf-A1) is a subclass of macrolide antibiotics isolated from *Streptomyces griseus* that serves as a specific and potent inhibitor of vacuolar-type H^+^-ATPases (V-ATPases) [11]. Baf-A1 was identified as the first specific V-ATPase inhibitor in the 1980s [12]. It has been used at an effective concentration in various cell types associated with many diseases. Previous reports showed that Baf-A1 exhibited potent antiviral activities against influenza A with IC50 values in the nanomolar range. This effect was mediated by inhibiting the activity of endosomal ATP-driven proton pumps [13]. Moreover, some studies suggest that Baf-A1 is an anti-autophagy [14], anti-tumorigenic, anti-parasitic, or anti-neurodegenerative drug [15]. Baf-A1 has been reported to inhibit endosomal acidification, which suppresses SARS-CoV-2 viral replication [16, 17]. Our previous study has demonstrated the antiviral efficacy of Baf-A1 against SARS-CoV-2 in Vero E6 cells and human angiotensin-converting enzyme 2 (hACE2) transgenic mice [17]. However, more comprehensive studies are needed to evaluate the clinical application of Baf-A1.

ACE2 is the entry receptor for SARS-CoV-2 [18]. hACE2 transgenic mouse models have been used for the studies of SARS-CoV-2 infection and the therapeutic screening of COVID-19 target candidates [19, 20]. However, this technique has disadvantages, such as cumbersome establishment [21]. Most recently, human chimeric lung mouse models have been established by grafting human fetal lung tissues under the skin or renal capsules of immunodeficient mice. These models establish the transplanted human fetal lung tissues, which have a typical vascularized, growing, and mature structure that closely resembling a normal adult lung [22]. Therefore, the xenografted human lung mouse model provides more clinically relevant data than hACE2 transgenic mouse models. Previous studies have demonstrated the value of using human lung xenograft mouse models in assessing the replication and infectivity of the Nipah virus [23], Zika virus, respiratory syncytial virus [24], and varicella-zoster virus [25]. Our laboratory also established a xenografted human lung mouse model for SARS-CoV-2 infection and drug screening.

In our previous study, Baf-A1 was used for SARS-CoV-2 infection in the hACE2 transgenic mouse model [17]. In this study, we provided an experimental basis for further investigations on the therapeutic effects of Baf-A1 against SARS-CoV-2 infection using the established human lung xenograft mouse model. Moreover, we explored whether Baf-A1 can reduce virus replication of SARS-CoV-2 variants for a more comprehensive evaluation of the antiviral capacity of Baf-A1.

## Materials and methods

### Cells, virus strains, and chemicals

African green monkey kidney Vero E6 (CL-0491) cells were obtained from Procell Life Science & Technology (China), and cultured in Dulbecco’s modified Eagle medium (DMEM, Sigma-Aldrich, D5796), supplemented with 10% fetal bovine serum (FBS, Invitrogen, 10,270), 50 U ml^−1^ penicillin and 50 µg ml^−1^ streptomycin, and maintained in a 37 ℃ incubator with 5% CO_2_.

The SARS-CoV-2 strain BetaCoV/Beijing/IME-BJ01/2020 was initially isolated by CFQ’s laboratory (Beijing, China) as described previously [26, 27]. The Beta variant GDPCC (CSTR: 16698.06.NPRC2.062100001) was isolated from an imported case in South Africa [28]. The Delta variant (CSTR:16698.06.NPRC6.CCPM—8-V-049-2105-6) was obtained from the National Pathogen Resource Center (NPRC), China. All viruses were propagated and titrated using a standard plaque assay in Vero E6 cells. All experiments with the SARS-CoV-2 virus were conducted in a biosafety level-3 laboratory.

Remdesivir and Baf-A1 were purchased from MedChemExpress (Monmouth Junction, New Jersey, USA).

### Human lung xenograft mouse model

Male NCG (NOD/ShiLtJGpt-Prkdcem26Cd52Il2rgem26Cd22) mice, weighing 22–29 g and bearing a fragment of the human fetal lung in the dorsal subcutaneous space, were kindly provided by Prof. Yong-Guang Yang from the First Hospital of Jilin University, Changchun, China. The mature pulmonary structure was developed in the xenografted fetal human lungs as previously described [23, 26].

### Measurement of viral titers

Vero E6 cells were inoculated in 96-well plates at 7 × 10^3^ cells per well and infected with SARS-CoV-2, Beta variant or Delta variant, MOI of 0.08, respectively. After incubation for the indicated times, the xenograft human lung tissues were isolated from NCG mice, homogenized, and serially diluted in DMEM medium. Ten-fold dilutions were added to the 96-well plates at a volume of 100 µl/well. After 2 h of infection, 100 µl DMEM containing 4% fetal bovine serum, 100 IU/mL penicillin and 100 µg/mL streptomycin were added to a final volume of 200 µl/well. Finally, the cells were incubated at 37 °C and 5% CO_2_ for 5 days. Viral titres were calculated as TCID50 using the Reed-Muench method.

### Cytotoxicity and antiviral activity assays

Cytotoxicity and antiviral activity assays were evaluated using the CCK-8 regent (Dojindo Molecular Technologies, Rockville, USA) according to the manufacturer’s instructions. Briefly, Vero E6 cells were inoculated in 96-well plates at 7 × 10^3^ cells per well and incubated at 37 °C and 5% CO_2_ for 24 h. After that, the medium was changed into phosphate-buffered saline (PBS). Subsequently, MEM containing 0, 0.016, 0.08, 0.4, 2, and 10 µM of the Baf-A1 was added to the cells and incubated for 2 h. Finally, the medium was removed, and 100 µL CCK8 (1 mg/mL in DMEM medium) was added to each well.

Baf-A1 was added 2 h prior to virus treatment to measure antiviral activity. The culture plates were incubated at 37 °C in a humidified 5% CO_2_/95% air incubator for 48 h. Following this step, the medium was removed, and 100 µL CCK8 (1 mg/mL in DMEM medium) was added into each well and incubated for 3 h at 37 °C. The optical density (O.D.) at the dual wave lengths of 450/630 nm was determined using a microplate reader (BIO-TEK EPOCH, USA). The results were transformed into a percentage of the controls. The dose-response curves were plotted using the GraphPad Prism 6 software.

### Real-time quantitative PCR (RT-qPCR) analysis

Vero E6 cells were seeded into 6-well plates at a density of 2 × 10^5^ cells/well for 24 h. Then, SARS-CoV-2, Beta, and Delta variants were infected at a multiplicity of infection (MOI) of 0.008. The QIAamp Viral RNA Kit (Qiagen, 52,906) was used to extract the viral RNA in the supernatant. Viral copy numbers were detected by absolute quantitative RT-qPCR methodology using the HiScript II One Step RT-qPCR SYBR Green Kit (Vazyme Biotech, Nanjing, China) on an ABI 7500 real-time PCR system (Applied Biosystems, CA, USA). The protocol for RT-qPCR was as follows: 50 °C for 15 min, 95 °C for 30 s, followed by 45 cycles at 95 °C for 10 s and 63 °C for 35 s. The specific primers used to detect the N and E genes were as follows:

- N gene Forward: 5′-GGGGAACTTCTCCTGCTAGAAT-3′;
- Reverse: 5′-CAGACATTTTGCTCTCAAGCT-3′;
- E gene Forward: 5′-CGATCTCTTGTAGATCTGTTCTC-3′.
- Reverse: 5′-ATATTGCATTGCAGCAGTACGCACA-3′.

The sequences of the TaqMan probe were as follows:

- N gene 5′-FAM-TTGCTGCTGCTTGACAGATT-TAMRA-3′.
- E gene 5′-FAM-ACACTAGCCATCCTTACTGCGCTTCG-BHQ1-3′.

The xenografted human lung tissue samples isolated from mice were collected and homogenized in PBS. Viral RNA was isolated and processed in the homogenate as described above.

### Crystal violet staining

Cell proliferation was examined using crystal violet staining as previously described [29]. Briefly, Vero E6 cells (2 × 10^5^ cells/well) were transferred into 6-well plates and incubated at 37 °C, 5% CO_2_ for 24 h. Vero E6 cells were pre-treated with Baf-A1 and Remdesivir for 2 h before virus administration. After incubation for 48 h, the cells were stained with crystal violet and fixed with 4% paraformaldehyde for 1 h. Finally, the stained cells were analyzed by microscopy, and representative images were captured using a digital camera.

### Animal experiments

As previously described, the xenografted fetal human lungs developed mature pulmonary structures [23]. Mice were randomly allocated into 4 experimental groups (n = 20). DMSO or SARS-CoV-2 was injected into the NCD mice under 10^5.5^ TCID50. Baf-A1 (0.1 mg/kg) or Remdesivir (15 mg/kg) was intraperitoneally added 2 h before SARS-CoV-2 infection. Then, NCD mice were treated with Baf-A1or Remdesivir on 1, 2, 3, 4, 5 dpi post-infection. Five NCD mice per group were sacrificed by cervical dislocation. Human lung tissues were isolated for viral titration, RT-qPCR analysis, RNA in situ hybridization assay, histopathological examination, and immunohistochemistry analysis.

All animals were housed at biosafety level 3 and given free access to standard pellet feed and water. All experiments were performed following the National Institute of Health Guide for the Care and Use of Laboratory Animals.

### RNA in situ hybridization assay

Lung tissues were fixed in a 4% paraformaldehyde solution containing 0.1% DEPC for 72 h. Tissues were heated for 1 h at 60 ˚C in an oven to dehydrate, deparaffinized in xylene for 10 min, and dehydrated in 100% ethanol for 5 min. After the sections were air-dried, RNA scope hydrogen peroxide solution was used to block the endogenous peroxidase for 7 min at room temperature and washed with distilled water twice for 5 min each. The hybridization was performed with a specific probe: 5′-DIG-ACTACAGCCATAACCTTTCCACATACCGCAGAC-DIG-3′. The DIG label was detected using an anti-DIG-HRP antibody. After incubation with DAB, images were captured by light microscopy, and the areal density was analyzed using Image pro plus 6.0 [26].

### Histopathological examination

Lung tissues were fixed in 4% paraformaldehyde solution for 7 days, paraffin-embedded, sectioned, and stained with hematoxylin and eosin (H&E) for assessing general histological structure according to standard protocols [30]. Images were captured by light microscopy and analyzed as previously described. To differentiate the lungs pathology, histology scores were calculated as previously described by grading the severity of damage in the bronchioles, alveoli, and blood vessels, and the total score was calculated. [31] The degree of damage was scored as follows: 0, normal structure; 1, mild infiltration, epithelial cell thickening, and congestion; 2, moderate epithelial cell death, infiltration, focal exudation, or widened alveolar septum; and 3, severe epithelial cell death, diffuse inflammatory cell infiltration, or collapse of the alveolar structure.

### Immunohistochemistry analysis

Lung tissues were fixed in 4% paraformaldehyde solution for 7 days, paraffin-embedded, and 3–4 µm thin sections were cut. Sections were defatted, rehydrated, and antigens retrieved using a citric acid (pH 6.0) antigen retrieval buffer. Next, the endogenous peroxidase activity was blocked using 3% hydrogen peroxide for 25 min at room temperature. After washing three times with PBS, the tissues were sealed for 30 min by adding 3% BSA. The sections were incubated overnight with primary antibodies against TNF-α, IL-1β, and ICAM-1 at 4˚C. On the next day, the sections were washed and incubated with HRP-conjugated secondary antibodies for 50 min, and 3,3′-diaminobenzidine (DAB) color-developing solution was added, followed by counterstaining with hematoxylin stain solution for nuclear staining. The final DAB-labelled images were captured by microscopy. The nucleus became blue after hematoxylin staining, and DAB-positive sections were stained brownish yellow. The images were converted into black and white, and a unified standard was established to identify positive signals. The integrated optical density (IOD) and pixel area of the cells were measured, and the areal density was calculated as the IOD/corresponding pixel area of the target cell.

### Statistical analysis

All measurements were made in triplicate, and all values are presented as mean ± SD. Statistical differences were compared with analysis of variance (ANOVA) followed by the Tukey test. P < 0.05 was considered statistically significant.

## Results

### Baf-A1 inhibits the replication of SARS-CoV-2 variant strains in Vero E6 cells

**Fig. 1:**
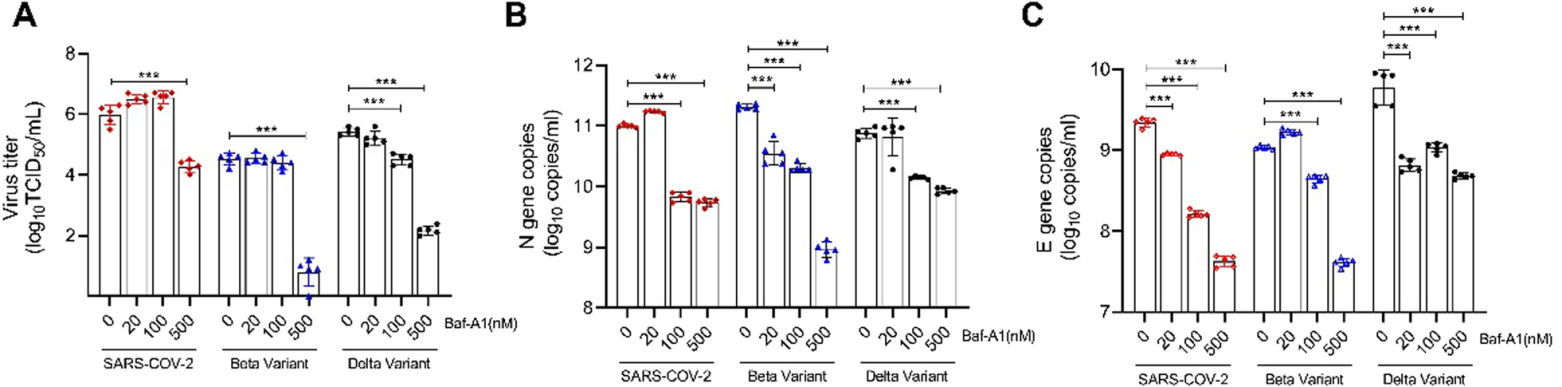
Baf-A1 inhibits SARS-CoV-2 replication in Vero E6 cells. Cells were infected with SARS-CoV-2 (0.008 MOI), Beta variant (0.008 MOI) or Delta variant (0.008 MOI) before treatment with different doses of Baf-A1. **A** Viral load in the infected Vero E6 cells. **B** RT-qPCR analysis of N gene copies after virus infection and treatment with different doses of Baf-A1. **C** RT-qPCR analysis of E gene copies after viral infection and treatment with different doses of Baf-A1. Three experiments were performed (n = 5 each group). ***p < 0.001

Baf-A1 inhibition of H^+^-ATPases has been shown to induce the acidification of endosomes, thus resulting in antiviral effects [11]. A previous study showed that Baf-A1 significantly reduces the yields of the SARS-CoV-2 virus in Vero E6 cells [17]. To further investigate the antiviral efficacy of Baf-A1 on variant strains, its effects on Beta and Delta variants were tested. TCID_50_ was used to show the number of live infectious viruses. The results showed that 500 nM Baf-A1 was effective against Beta and Delta variants. In comparison, a significant diminution in SARS-CoV-2 replication was observed at the concentration of 100 nM (Fig. 1A). RT-qPCR is a reliable test for testing the genome of SARS-CoV-2, and the N and E genes are important targets for differentiating SARS-CoV-2 from other coronaviruses. RT-qPCR analysis was used to measure the copy number of the virus gene. The results showed that Baf-A1 decreased the N and E gene copies of SARS-CoV-2 and its variants (Fig. 1B, C).

### Baf-A1 improves the viability of Vero E6 cells after infection with SARS-CoV-2 variant strains

**Fig. 2:**
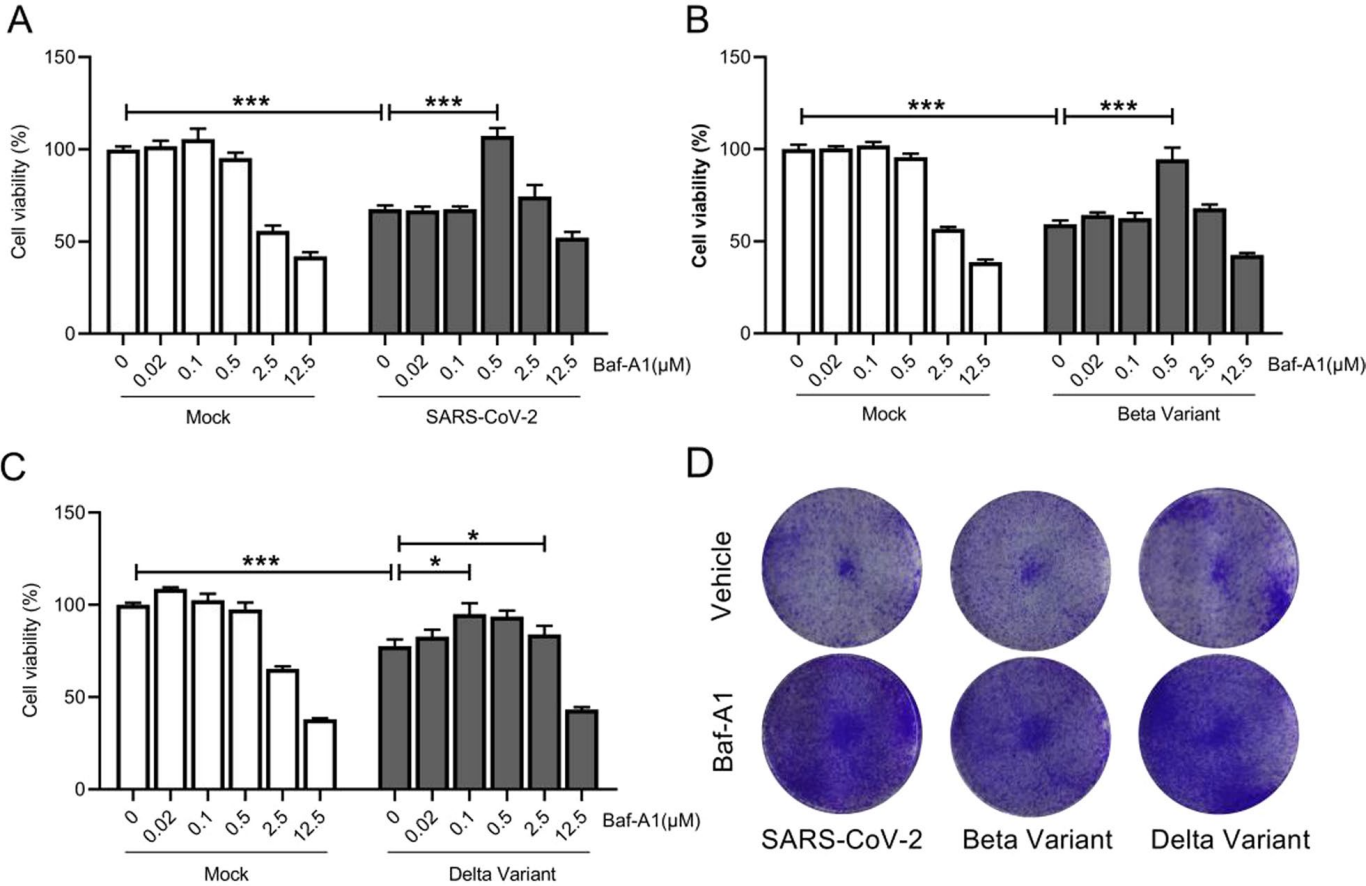
Baf-A1 improves the viability of Vero E6 cells after infection with SARS-CoV-2 variant strains. **A** Viability of Vero E6 cells treated with different doses of Baf-A1 after infection with SARS-CoV-2 (0.008 MOI) for 48 h. **B** Viability of Vero E6 cells treated with different doses of Baf-A1 and after infection with Beta variant (0.008 MOI) for 48 h. **C** Viability of Vero E6 cells treated with different doses of Baf-A1 and after infection with Delta variant (0.008 MOI) for 48 h. **D** Crystal violet staining assays of Vero E6 cells treated with Baf-A1 (0 and 500 nM) after infection (0.008 MOI) for 48 h. Three experiments were performed (n = 5 each group). *p < 0.05, **p < 0.01, ***p < 0.001

Vero E6 cell viability was determined using CCK-8 assays. SARS-CoV-2 and its variants resulted in significant damage to Vero E6 cell; 500 nM Baf-A1 increased the survival rate of cells infected with SARS-CoV-2 and Beta variants by 50 and 30%, respectively (Fig. 2A, B), while, Baf-A1 showed a minor effect after Delta variant infection at 100 nM and 500 nM (Fig. 2C). At these concentrations, there was no effect on cell activity. However, when the amount of Baf-A1 reached 2.5 µM, it produced a toxic effect. Correspondingly, crystal violet staining assays showed that SARS-CoV-2 and its variants cause significant damage to Vero E6 cells after 72 h post-infection. In summary, 500 nM Baf-A1 improves cell proliferation in Vero E6 cells after infection (Fig. 2D).

## Baf-A1 inhibits the replication of SARS-CoV-2 in xenografted human lung tissues

**Fig. 3:**
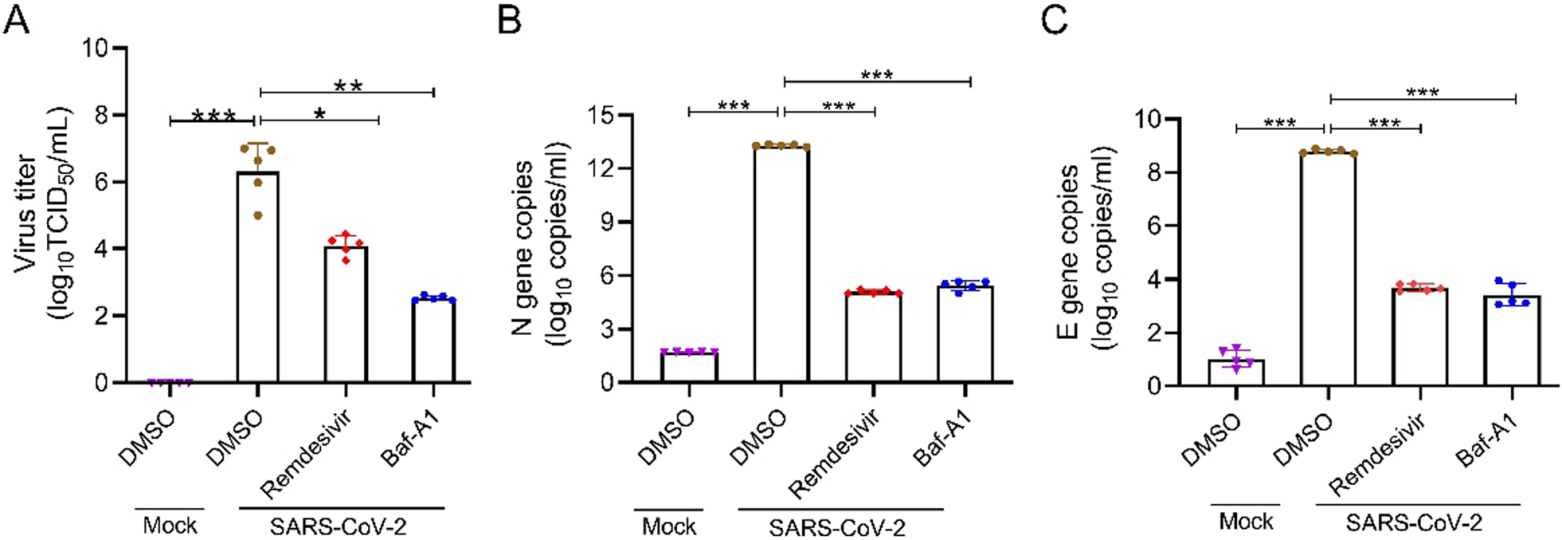
Baf-A1 inhibits SARS-CoV-2 replication in xenografted human lung tissues vehicle. DMSO or SARS-CoV-2 was injected into the NCG mice engrafted with human lung tissues under 10^5.5^ TCID50. Baf-A1 (0.1 mg/kg) or Remdesivir (15 mg/kg) was intraperitoneally added 2 h before SARS-CoV-2 infection. Then, NCD mice were treated with Baf-A1or Remdesivir on 1, 2, 3, 4, 5 dpi post infection. Five NCD mice per group were sacrificed by cervical dislocation and human lung tissues were isolated for virus titration and RT-qPCR. **A** Viral titers of the SARS-CoV-2-infected human lung tissues from Baf-A1 or Remdesivir treated mice on 5 dpi. **B**, **C** RT-qPCR analysis of SARS CoV-2 N (**B**) and E gene (**C**) copies in the Mock, SARS-CoV-2, Baf-A1 or Remdesivir treated human lungs on 5 dpi

The xenografted human lung mouse model was previously used to study SARS-CoV-2 infection and replication in human lung tissues and induce pneumonia-like pathological changes [26]. Remdesivir has been approved for the treatment of SARS-CoV-2 [32]. Therefore, we determined whether Baf-A1 confers protection to human lungs after SARS-CoV-2 infection and treatment with remdesivir was used as a positive control drug. The results showed that the yields of SARS-CoV-2 were markedly reduced in lung tissues, suggesting that the viral replication had been suppressed by Baf-A1 (Fig. 3A). RT-qPCR analysis revealed that SARS-CoV-2 N and E gene copies continued to increase in lung tissues (Fig. 3B, C).

**Fig. 4:**
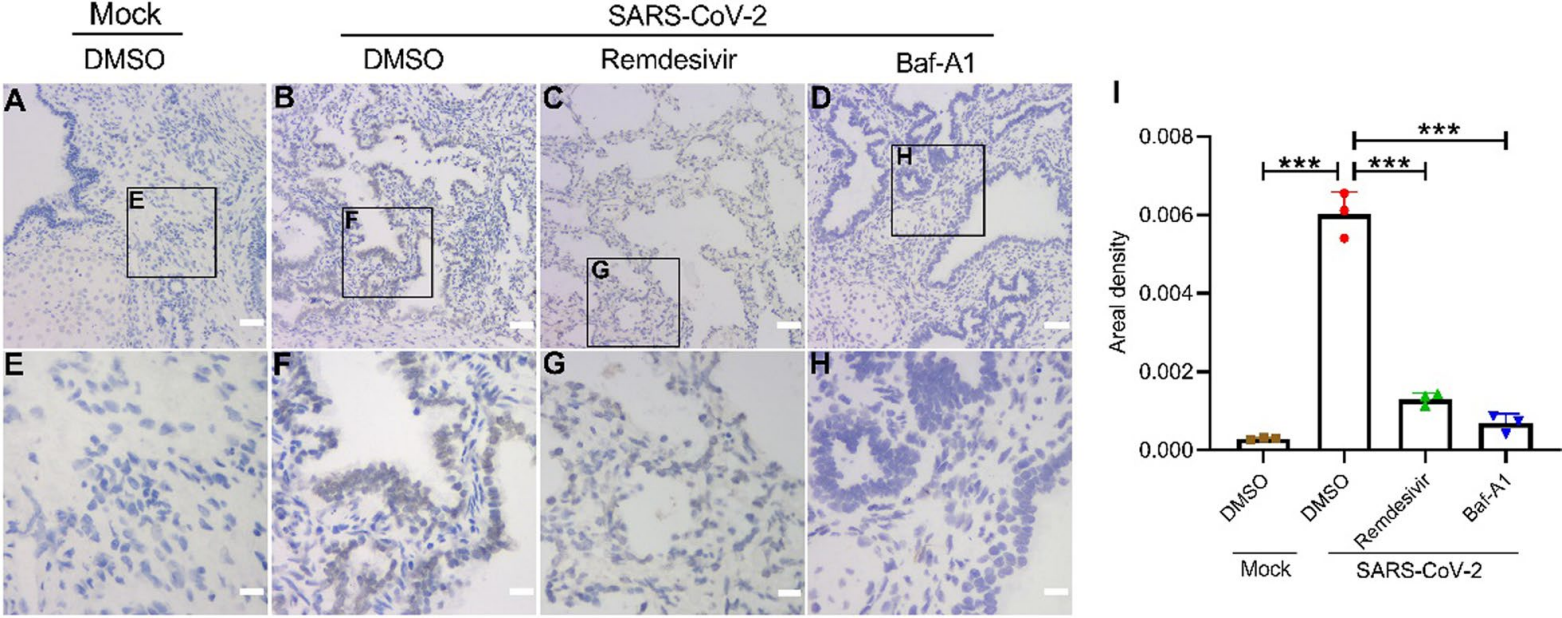
In situ hybridization analysis of SARS-CoV-2 RNA in human lung tissues treated with Mock (**A**, **E**), DMSO (**B**, **F**), Baf-A1 (**C**, **G**) or Remdesivir (**D**, **H**) after SARS-CoV-2 infection on 5 dpi. **I** The areas (Mean Density) were measured as described in the experimental procedures. Scale bar of A, B, C, D = 50 μm; Scale bar of E, F, G, H = 10 μm. Data were expressed as mean ± SEM from three independent experiments. *p < 0.05, **p < 0.01, ***p < 0.001

In addition, the viral RNA was detected by in situ hybridization. The results showed that hybridization signals were reduced in the lung tissues of Remdesivir or Baf-A1-treated mice (Fig. 4). Baf-A1 has the same effect as Remdesivir in suppressing SARS-CoV-2 replication in the xenografted human lung mouse model. More importantly, 0.1 mg/kg Baf-A1 treatment for 7 d does not appear to have adverse effects on mice, and no alterations in the body weight were observed.

### Baf-A1 ameliorates pneumonia in xenografted human lung tissues

**Fig. 5:**
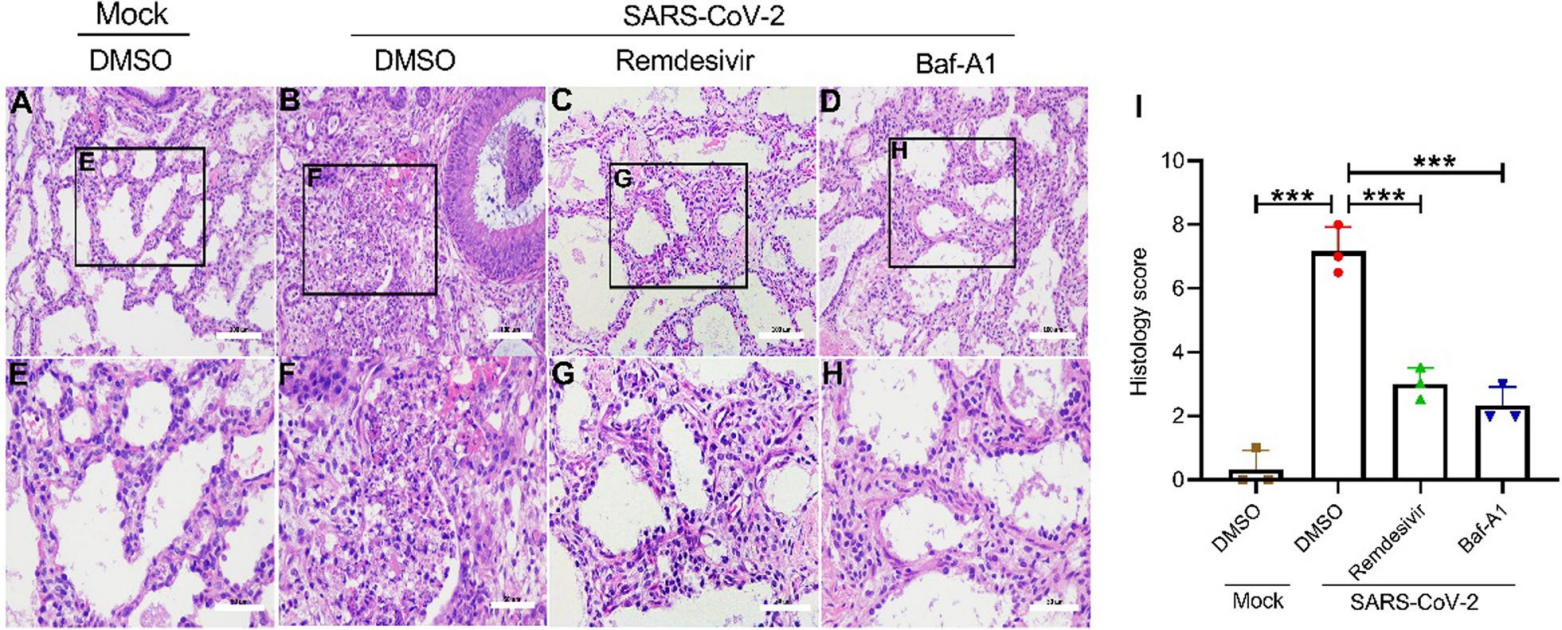
Baf-A1 ameliorates pneumonia in xenografted human lung tissues. Human lung sections were stained with H&E as described in the experimental procedures. **A**, **E** Images of H&E-stained Mock tissue; **B**, **F** Images of H&E-stained tissues with SARS-CoV-2 infection. The lung tissue structure was abnormally severe, with exudation of inflammatory cells into the perivascular tissues (yellow arrows), and the bronchial epithelium was hyperplastic (red arrows); **C**, **G** Images of H&E-stained tissues with Remdesivir treatment; **D**, **H** Images of H&E-stained tissues with Baf-A1 treatment. The alveolar structure is relatively clear, and the fibrous tissue hyperplasia can be seen in some areas (black arrows). **I** Histological score based on H&E staining indicating pathological lesions in lung tissues from Mock-infected, and SARS-CoV-2-infected mice treated with DMSO, remdesivir or Baf-A1. The score used for the measurement of damage is detailed in the methods section. Scale bar of A, B, C, D = 100 μm; Scale bar of E, F, G, H = 50 μm. Data were expressed as mean ± SEM from three independent experiments. *p < 0.05, **p < 0.01, ***p < 0.001

It has been reported that SARS-CoV-2 infection causes severe damage to the xenografted human lung mouse model, manifesting with diffuse inflammatory infiltration of granulocytes and induced pneumonia-like pathological changes [26]. The histopathological examination showed that the presence of pneumonia-associated pathological changes in SARS-CoV-2-infected xenografted human lung tissues on day 5 post-SARS-CoV-2 infection, including necrosis and abscission of the bronchiolar endothelial cells, peribronchiolar and perivascular infiltration of segmented granulocytes and alveolar septum thickening with inflammatory exudation (Fig. 5B). After treatment with Baf-A1 and Remdesivir, these pathological changes were ameliorated in human lung tissues (Fig. 5C, D). The mice’s body weights were not affected by either SARS-CoV-2 infection or drug treatment (data not shown).

**Fig. 6:**
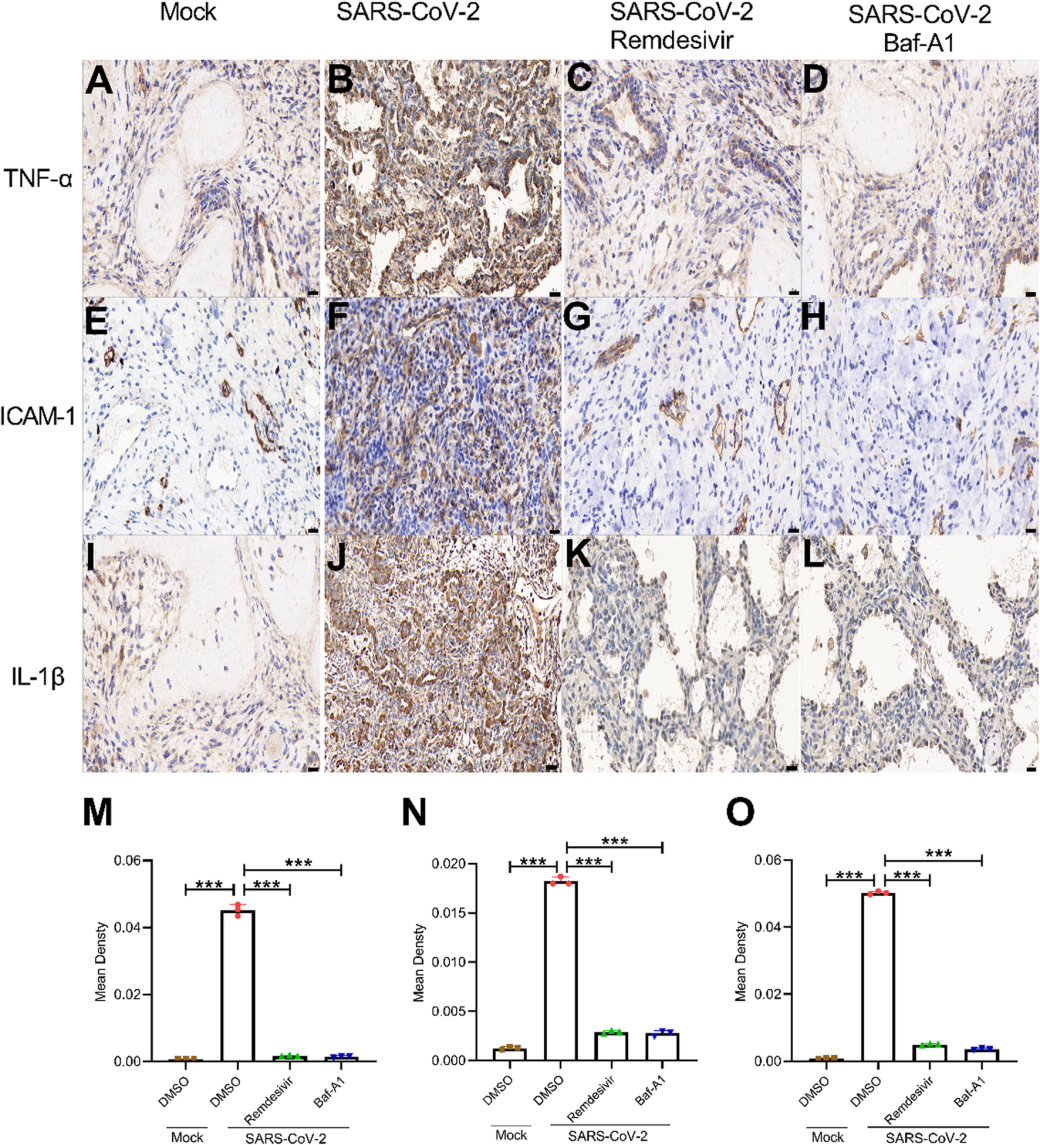
Immunohistochemistry staining of TNF-α, ICAM-1 and IL-1β in human lung tissues treated with Mock (**A**, **E**, **I**), DMSO (**B**, **F**, **J**), Baf-A1 (**C**, **G**, **K**) or Remdesivir (**D**, **H**, **L**) after SARS-CoV-2 infection on 5 dpi. The AREA (Mean Density) of TNF-α (**M**), ICAM-1 (**N**), IL-1β (**O**) were measured as described in the experimental procedures. Scale bar = 20 μm. Data were expressed as mean ± SEM from three independent experiments. *p < 0.05, **p < 0.01, ***p < 0.001

To investigate the host responses following SARS-CoV-2 infection in the human lung and measure the efficacy of Baf-A1, the expression levels of several cytokines and chemokines were determined in the homogenates of human lung xenografts (Fig. 6). The expression of human cytokines or chemokines was primarily associated with SARS-CoV-2 infection in human epithelial and endothelial cells. SARS-CoV-2 infection in human lung increased the expression of several cytokines and chemokines, including TNF-a, ICAM-1, and IL-1b (Fig. 6B, F, J). However, the expression levels of these cytokines and chemokines significantly decreased after Baf-A1 (Fig. 6 C, G, K) and Remdesivir (Fig. 6D, H, L) treatment.

## Discussion

In the present study, Baf-A1 significantly inhibited SARS-CoV-2 replication in vitro and human lung xenografts. In Vero E6 cells, Baf-A1 also improved cell proliferation, which was consistent with our previous findings [26]. Furthermore, our results showed that Baf-A1 has a greater potency against the variant strains (Beta variant and Delta variant), indicating its broad-spectrum tolerability and high sensitivity to strains. The significant differences in the effectiveness of Baf-A1 on the variant strains may be attributed to the combination of key mutations, giving the spike protein a higher binding affinity to ACE-II [33]. Thus, more studies are required to assess the variants fully.

It is generally accepted that SARS-CoV-2 can infect specific mouse models, including hACE2-transgenic mice and Golden hamsters [34]. Our previous study showed that chloroquine and Baf-A1 could reduce viral replication in lung tissues and alleviate viral pneumonia in the hACE2 transgenic mouse model [26]. To further verify the anti-SARS-Cov-2 value of Baf-A1, we used a human lung xenograft mouse model, a primary, translational clinical tool closer to the clinical model of COVID-19 lung infection. The safety of Baf-A1 in animals is worthy of our concern. A 0.1 mg/kg Baf-A1 treatment for 7 d does not appear to show alterations in body weight. However, its safety needs further verified on multiple models such as beagle and rabbit. The viral titer and quantitative fluorescence-based reverse transcription polymerase chain reaction are important assays for researchers to study infectious diseases. It was found that Baf-A1 reduced the virus replication of SARS-CoV-2 and its variants in both ways. A deficiency of leukocytes and NK cells resulted in poor adaptive immune responses in NCG mice [23]. However, its innate immune response could still be effective based on the presence of macrophages and granulocytes.

Moreover, our results also showed the positive signals of inflammatory factors, such as TNF-a, ICAM-1, and IL-1b, in the xenografted human lung tissue after SARS-CoV-2 infection or drug treatment. Notably, Baf-A1 and remdesivir were equally effective in reducing the presence of these inflammatory factors. However, due to limited clinical lung resources, the number of NGG mice can only support the performance of SARS-CoV-2 experiments. Therefore, the related studies on SARS-CoV-2 variants will be further explored as conditions permit.

Our results confirmed that Baf-A1 might be useful as a spectral antiviral agent against SARS-CoV-2 and its variants. The antiviral effectiveness of Baf-A1 is almost equal to that of remdesivir, both in cells and lung tissues. According to our previous study, the effective concentration of Baf-A1 was defined as 0.1 mg/kg in the human lung xenograft mouse experiments, and mice’s body weights were not affected. Nevertheless, due to the inherent cellular toxicity of Baf-A1, more safety trials are needed before further development of Baf-A1.

## Data Availability

Source data in this work are available at the GitHub repository: https://github.com/1502006/Bafilomycin.git.

## Abbreviations

SARS-CoV-2: Severe Acute Respiratory Syndrome Coronavirus-2
VOC: Variants of Concern
Baf-A1: Bafilomycin A1
WHO: World Health Organization
TNF-α: Tumor necrosis factor-α
ICAM-1: Intercellular cell adhesion molecule-1
IL-1β: Interleukin-1 beta
